# Transmissibility of H-Type Bovine Spongiform Encephalopathy to Hamster PrP Transgenic Mice

**DOI:** 10.1371/journal.pone.0138977

**Published:** 2015-10-14

**Authors:** Hiroyuki Okada, Kentaro Masujin, Kohtaro Miyazawa, Takashi Yokoyama

**Affiliations:** National Institute of Animal Health, National Agriculture and Food Research Organization (NARO), Tsukuba, Ibaraki, Japan; Creighton University, UNITED STATES

## Abstract

Two distinct forms of atypical bovine spongiform encephalopathies (H-BSE and L-BSE) can be distinguished from classical (C-) BSE found in cattle based on biochemical signatures of disease-associated prion protein (PrP^Sc^). H-BSE is transmissible to wild-type mice—with infected mice showing a long survival period that is close to their normal lifespan—but not to hamsters. Therefore, rodent-adapted H-BSE with a short survival period would be useful for analyzing H-BSE characteristics. In this study, we investigated the transmissibility of H-BSE to hamster prion protein transgenic (TgHaNSE) mice with long survival periods. Although none of the TgHaNSE mice manifested the disease during their lifespan, PrP^Sc^ accumulation was observed in some areas of the brain after the first passage. With subsequent passages, TgHaNSE mice developed the disease with a mean survival period of 220 days. The molecular characteristics of proteinase K-resistant PrP^Sc^ (PrP^res^) in the brain were identical to those observed in first-passage mice. The distribution of immunolabeled PrP^Sc^ in the brains of TgHaNSE mice differed between those infected with H-BSE as compared to C-BSE or L-BSE, and the molecular properties of PrP^res^ in TgHaNSE mice infected with H-BSE differed from those of the original isolate. The strain-specific electromobility, glycoform profiles, and proteolytic cleavage sites of H-BSE in TgHaNSE mice were indistinguishable from those of C-BSE, in which the diglycosylated form was predominant. These findings indicate that strain-specific pathogenic characteristics and molecular features of PrP^res^ in the brain are altered during cross-species transmission. Typical H-BSE features were restored after back passage from TgHaNSE to bovinized transgenic mice, indicating that the H-BSE strain was propagated in TgHaNSE mice. This could result from the overexpression of the hamster prion protein.

## Introduction

Bovine spongiform encephalopathy (BSE), a type of prion disease or transmissible spongiform encephalopathy, is a fatal and progressive degenerative central nervous system (CNS) disorder in cattle. BSE was first identified in the United Kingdom in 1986 [1], and spread to European as well as North American countries and Japan, and has affected more than 190,000 cattle worldwide. The disease is characterized by spongiform changes and accumulation of an abnormal isoform of prion protein (PrP^Sc^) that is thought to harbor a post-translational conformational change of the normal, host-encoded cellular prion protein (PrP^C^) in the CNS of affected hosts [2]. BSE in cattle has been classified into at least three distinct strains, including classical (C-) and H- and L-type atypical BSEs based on the molecular features of the protease-resistant PrP^Sc^ (PrP^res^) identified by western blotting (WB) [3–6]. The origins of BSEs are still unclear; however, based on epidemiological data, C-BSE is probably caused by the consumption of BSE-contaminated meat and bone meal from C-BSE-infected carcasses or scrapie-infected ruminants [7, 8]. Around 90 atypical BSE cases have been detected worldwide over the past 8 years, which may represent sporadic forms that arose spontaneously [6, 9].

BSE is transmissible in a wide range of host species. The transmission of H-BSE to cattle [10–15], transgenic mice overexpressing bovine PrP [13, 16, 17], tg338 transgenic mice expressing the V_136_R_154_Q_171_ allele of ovine PrP [16], and wild-type mice [18–20] has been demonstrated experimentally. However, transmission was less efficient to an TgOvPrP4 ovine transgenic mouse model expressing the A_136_R_154_Q_171_ allele [21] and to Syrian hamsters [22], suggesting the existence of a species barrier. Although wild-type mice are susceptible to H-BSE, it requires a long period of time nearly the entire lifespan (< 600 days) even after adaptation [18–20]. Therefore, mouse models of H-BSE with short survival periods can elucidate the characteristics and origin of H-BSE. Transgenic mice overexpressing heterologous PrP genes can serve as rodent models for investigating the BSE prion species barrier. Although C-BSE from cattle is inefficiently transmitted to hamsters and to transgenic mice overexpressing hamster PrP in their neurons (TgHaNSE), mouse-passaged C-BSE was permissive to these animals in experimental challenges [23, 24]. On the other hand, L-BSE from cattle can be transmitted to hamsters and TgHaNSE mice [22, 23, 25]. In the present study, we evaluated the transmissibility of H-BSE to TgHaNSE mice and characterized the spectrum of H-, C-, and L-BSE phenotypes in TgHaNSE mice.

## Materials and Methods

### Ethics statement

Animal experiments were carried out in strict accordance with the regulations outlined in the Guide for the Care and Use of Laboratory Animals of the National Institute of Animal Health and the Guidelines for Proper Conduct of Animal Experiments, 2006 by the Science Council of Japan. Procedures involving animals were approved by the Institutional Animal Care and Use Committee at the National Institute of Animal Health (approval ID: 10–005, 11–008, and 13–005), with all possible effort made to minimize the pain and discomfort of each animal in accordance with the Guidelines for Animal Transmissible Spongiform Encephalopathy Experiments of the Ministry of Agriculture, Forestry, and Fisheries of Japan. All intracerebral inoculations were performed under sevoflurane anesthesia.

### BSE transmission to TgHaNSE mice

Female TgHaNSE mice (*n* = 5; 3 weeks old) were intracerebrally inoculated with 20 μl of 10% brainstem homogenate (weight/volume) from cattle experimentally infected with H-BSE [13]. The original H-BSE isolate was obtained from the Canadian Food Inspection Agency (Lethbridge, Canada) [26]. PrP^Sc^-positive brains of first-passage TgHaNSE mice were used for subsequent passages to obtain the TgHaNSE mouse-adapted H-BSE prion. The expression level of hamster PrP in the brain of TgHaNSE mice (provided by Dr. B. Chesebro) was approximately 4–5 times higher than that of Syrian hamsters [27].

To compare biological and biochemical properties of C- and L-BSE prions, brain homogenates prepared from C-BSE passaged once in ICR mice or Japanese L-BSE (named as BSE/JP24) from National Institute of Infectious Diseases (Tokyo, Japan) [28] were injected into TgHaNSE mice (Table 1). C-BSE from cattle was not transmissible to TgHaNSE mice unless it was passaged once in ICR mice with a survival period of 153 days [23]. Therefore, mouse-passaged C-BSE was used in this study. Pathological and molecular characteristics of C- and L-BSE transmitted to TgHaNSE mice have been described elsewhere [23, 25].

**Table 1:** Characteristics of monoclonal antibodies used in this study.

Clone	Epitope location	PrP sequence numbering	Species reactivity	Source
3F4	109-MKHM-112	Hamster	Hamster	Millipore (Billerica, MA, USA)
T2	discontinuous unknown (132–156)	Mouse	Hamster, mouse, cattle	Shimizu et al. [29]
6H4	144-DYEDRYYRE-152	Human	Hamster, mouse, cattle	Prionics (Schlieren, Switzerland)
4E10	147-RYYRENMYRYPN-158	Mouse	Mouse	Iwamaru et al. [31]
SAF84	175-RPVDQY-180	Cattle	Hamster, mouse, cattle	SPI-bio (Montigny le Bretonneux, France)

### Monoclonal antibodies (mAbs)

Characteristics of the five primary antibodies used in this study are summarized in Table 1. The mAbs T2 [29], 6H4, and SAF84 reacted with bovine, hamster, and mouse PrP, respectively; 3F4 bound to hamster PrP, but did not react with bovine or mouse PrP [30]; and 4E10 recognized mouse but not hamster or bovine PrP [31].

### Histological analysis

At necropsy, the left hemisphere and selected tissues—including lymphoid organs—were fixed in 10% buffered formalin containing 10% methanol. Formalin-fixed tissue specimens were immersed in 98% formic acid for 60 min to reduce the infectivity, then embedded in paraffin, sectioned, and stained with hematoxylin and eosin (HE) for histological evaluation or processed for PrP^Sc^ immunohistochemistry (IHC).

### PrP^Sc^ immunohistochemistry

After appropriate epitope retrieval by hydrate autoclaving in 10 mM citrate buffer (pH 6.0) at 121°C for 3 min or with a combination of enzymatic and chemical treatments [32], sections were incubated with mAbs 3F4 or SAF84. An indirect horseradish peroxidase polymer system (Histofine Simple Stain MAX-PO [M]; Nichirei, Tokyo, Japan) with 3, 3'-diaminobenzedine tetrachloride as the chromogen was used for visualization. Sections were lightly counterstained with Mayer’s hematoxylin.

### Western blotting (WB)

The right half of each brain was digested with 40 μg/ml PK (Roche Diagnostics, Indianapolis, IN, USA). Tissue samples were dissolved in sodium dodecyl sulfate polyacrylamide gel electrophoresis sample buffer and assayed using standard WB procedures. PrP^res^ was detected with mAbs 3F4, 4E10, T2, 6H4, or SAF84 and a chemiluminescent substrate (SuperSignal; Pierce Biotechnology, Rockford, IL, USA) as previously described [25]. After PK treatment, some samples were deglycosylated with N-glycosidase F (PNGase F; New England Biolabs, Ipswich, MA, USA) according to the manufacturer’s instructions.

### Back passage of H-BSE in TgHaNSE mice to bovinized PrP transgenic (TgBoPrP) mice

To compare disease phenotypes and molecular characteristics of the H-BSE inoculum before and after passage in TgHaNSE mice, brain homogenates from diseased first-passage TgHaNSE mice were intracerebrally injected back into TgBoPrP mice. The susceptibility of these mice (kindly provided by Dr. S.B. Prusiner [33]) to H-BSE has been previously confirmed [13].

## Results

### Transmission of H-BSE from cattle to TgHaNSE mice

None of the TgHaNSE mice injected with the H-BSE prion exhibited typical signs of the disease at first passage (Table 2). Four of five mice were sacrificed at the end of their lifespan of > 700 days. The remaining mouse was found dead 852 days post-inoculation. Histopathological and immunohistochemical analyses were not possible owing to severe autolysis of the brain.

**Table 2:** Transmissibility of BSE prions to TgHaNSE mice.

Inoculum	Passage	Survival period, dpi (mean ± SD)	Number of diseased/inoculated mice	Frequency of PrP^Sc^-positive brains
C-BSE	P1	160.3 ± 1.6	6/6	6/6
L-BSE	P1	567*, 853*, 860	0/3	2/3
	P2	148.7 ± 7.5	6/6	6/6
H-BSE	P1	787.0 ± 85.4	0/5	3/5
	P2	223.4 ± 7.7	14/14	14/14
	P3	238 ± 2.5	5/5	5/5

dpi, days post-inoculation; SD, standard deviation; BSE, bovine spongiform encephalopathy; C-BSE, classical BSE; L-BSE, L-type BSE; H-BSE, H-type BSE; PrP^Sc^, disease-associated prion protein; P1/P2/P3, passage 1/2/3.

*PrP^Sc^-positive brains.

After one passage of H-BSE from cattle in TgHaNSE mice, subsequent transmission to the mice resulted in shortened mean survival periods, with mice showing signs of prion disease. A third passage did not further reduce the survival period (Table 1).

### Histopathology

Lesion profiles based on average scores for histological vacuolation in nine neuroanatomical regions of coronal HE-stained brain sections [34] from TgHaNSE mice infected with the three different BSE agents were not obtained for TgHaNSE mice, because vacuoles were detected only in the reticular formation of the medulla, midbrain tegmentum of the brainstem, and in vestibular and thalamic nuclei, but not in other brain areas.

### Distribution of PrP^Sc^ deposits in the brain

**Fig 1:**
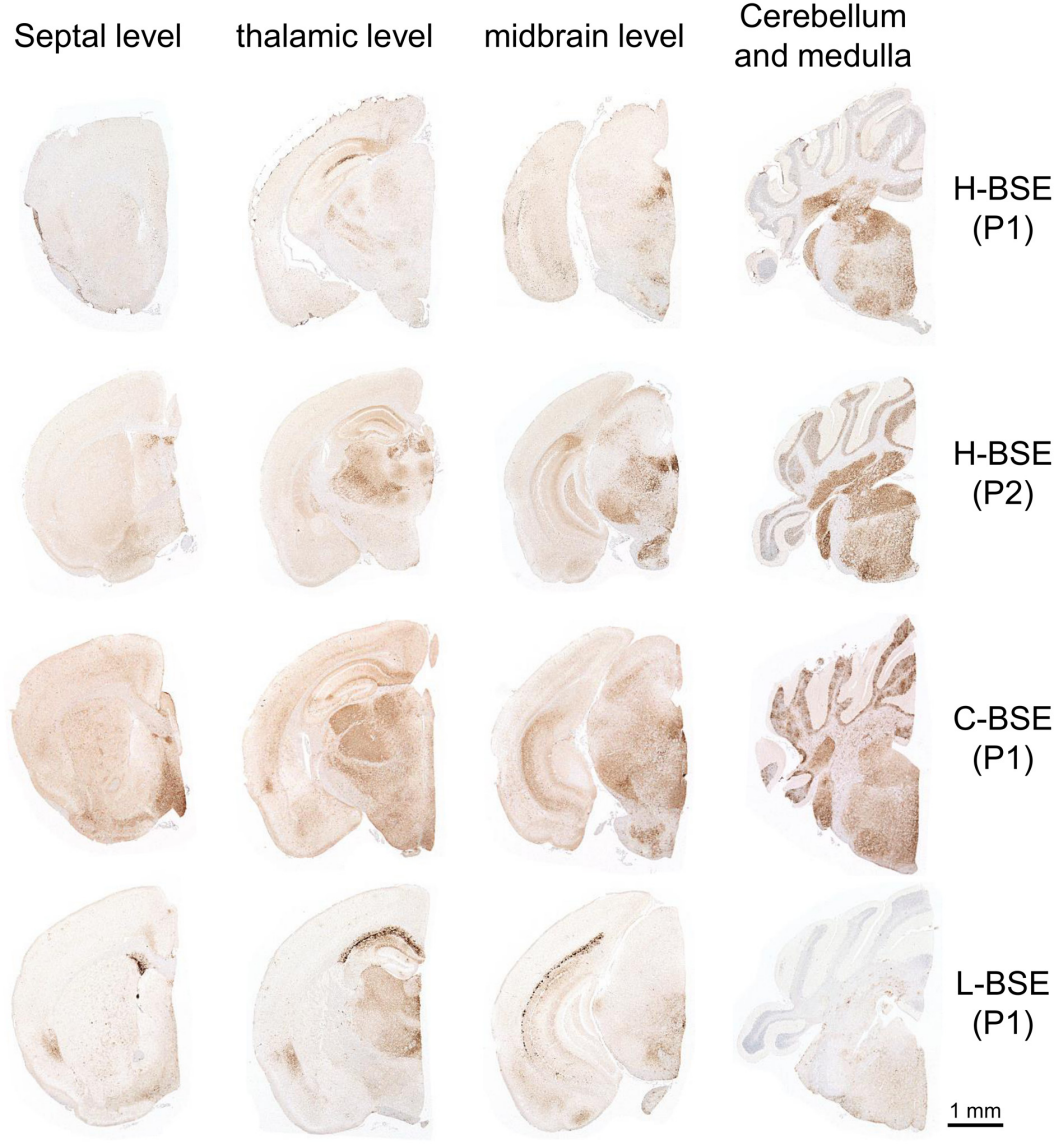
Neuroanatomical PrP^Sc^ distribution patterns in the brains of TgHaNSE mice infected with H-BSE, mouse-passaged C-BSE, or L-BSE. Representative images of coronal brain sections are shown. From left to right: septal level, hippocampus and thalamic level, midbrain, and medulla with cerebellum. PrP^Sc^ labeled with the mAb 3F4 was mainly distributed in the brainstem, thalamus, and hippocampus of TgHaNSE mice infected with H-BSE, but was deposited throughout the brain of mice infected with C-BSE. In L-BSE infected mice, PrP^Sc^ immunoreactivity was dense; the protein formed plaques in the periventricular and subcallosal regions. P1 and P2, first and second passage, respectively.

TgHaNSE mice infected with H-BSE showed positive PrP^Sc^ immunoreactivity at first and subsequent passages by IHC with the mAb 3F4, which reacts with hamster PrP (Fig 1). Three of four first-passage TgHaNSE mice infected with H-BSE had minimal PrP^Sc^ deposits in the brain that mostly consisted of fine punctate or coarse particles with compact aggregates in some areas of the brain, such as the medulla, midbrain tegmentum, raphe nuclei, red nucleus, cerebellar nuclei, hypothalamus, thalamus, habenular nucleus, and diagonal band of Broca (Fig 1). Fewer of the fine punctate PrP^Sc^ along with glia-associated stellate-type PrP^Sc^ were detected in cerebral and cerebellar cortices, striatum, thalamus, and the reticular formation of the brainstem. Perineuronal PrP^Sc^ deposits were common in the large neurons of the raphe nuclei and red nucleus, but were less common in other brain regions. In the cerebellar cortex, coarse particulate PrP^Sc^ accumulated in the granular layer. Additionally, a streak-like form of PrP^Sc^ was locally detected in the molecular layer of the cerebellum.

PrP^Sc^ deposits in the brains of second- and third-passaged mice infected with H-BSE were widespread in the hippocampus, thalamus, midbrain tegmentum, and molecular layers of the cerebellum as compared to those observed in first-passage mice (Fig 1). There were no obvious differences in the distribution or severity of PrP^Sc^ accumulation between second- and third-passage mice. In addition, positive PrP^Sc^ immunoreactivity was detected in the spinal cord, trigeminal and dorsal root ganglia, retina, optic nerve, inner ear (vestibulocochlear nerve/acoustic nerve), and muscle bundles or skeletal muscle fibers (S1 Fig).

**Fig 2:**
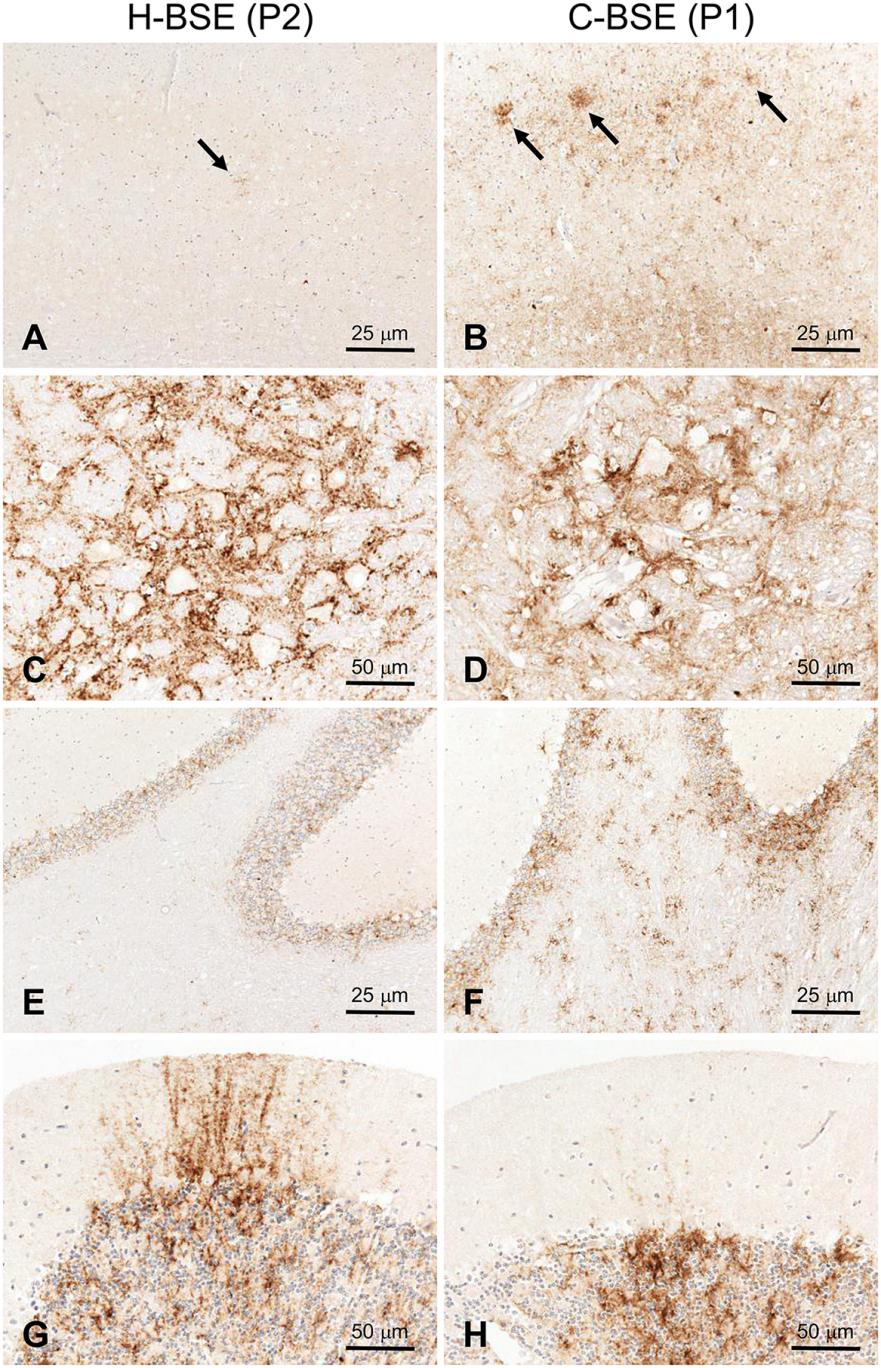
Features and types of PrP^Sc^ in TgHaNSE mice infected with H-BSE or mouse-passaged C-BSE. (A, B) Stellate-type PrP^Sc^ deposits (arrows) were observed in the cerebral cortex of H-BSE-infected mice (A), whereas particulate, punctuate, and stellate-type PrP^Sc^ were distributed in the cortex of C-BSE-infected mice (B). (C and D) Particulate (C) and fine punctuate (D) PrP^Sc^ immunoreactivity was observed in the red nuclei of the midbrain. (E–H) In the cerebellum, PrP^Sc^ immunoreactivity was particulate in the granular layer (E) and streaked in the molecular layer of H-BSE infected mice (G). In contrast, PrP^Sc^ was less prevalent in the molecular layer of C-BSE-infected mice; however, coarse particulate and stellate-type PrP^Sc^ accumulation was detected in the granular layer of cerebellar cortex and in the cerebellar medulla, respectively (F and H). P1 and P3, first and third passage, respectively.

Although targeted regions were similar between TgHaNSE mice infected with C-BSE and with H-BSE, PrP^Sc^ was more widely distributed in the brain of those infected with C-BSE (Fig 1). In the cerebral cortex, PrP^Sc^ was mainly deposited in a laminar pattern in layer 5. In addition, coarse particulate and stellate-type PrP^Sc^ were diffusely detected in the granular layer and medulla, respectively, of the cerebellum (Fig 2). In contrast, PrP^Sc^ distribution patterns of L-BSE differed from those of H-BSE and C-BSE. The most conspicuous PrP^Sc^ pattern in L-BSE consisted of amyloid plaque-like deposits lining the subcallosal and periventricular regions (Fig 1). PrP^Sc^ accumulation was less common in most brain areas of TgHaNSE mice infected with L-BSE as opposed to H- and C-BSE.

### Molecular features of PrP^res^ are similar between TgHaNSE mice infected with H-, C-, and L-BSE

**Fig 3:**
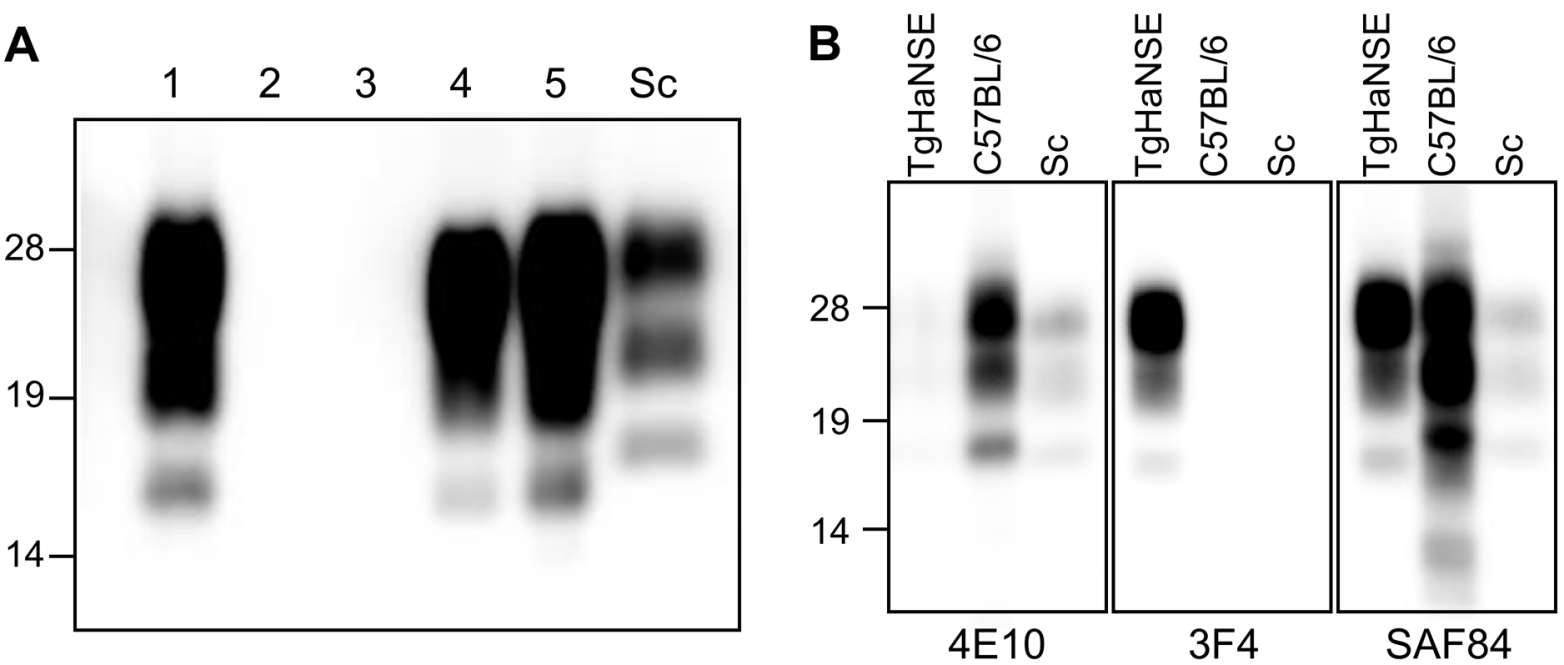
Western blot analysis of brains from first-passage, H-BSE-infected TgHaNSE mice. (A) Three of five mice showed positive immunoreactivity using the mAb T2. (B) PrP^res^ expression in the brains of TgHaNSE or C57BL/6 mice infected with H-BSE, as detected using mAbs 4E10, 3F4, and SAF84. Sc, mouse-adapted Obihiro scrapie control strain. Molecular markers are shown to the left (kDa).

Positive signals were detected by conventional WB analysis in only three of five first-passage H-BSE-infected mice (Fig 3A). The mAb 3F4 reacted only with PrP^res^ from the brains of TgHaNSE mice infected with H-BSE, and not with those of wild-type ICR mice infected with either H-BSE or mouse-adapted Obihiro-strain scrapie. The mAb 4E10 showed the opposite results. The mAb SAF84 yielded positive results for all samples but lacked the additional 10–12-kDa band that was detected in C57BL/6 mice infected with H-BSE (Fig 3B).

**Fig 4:**
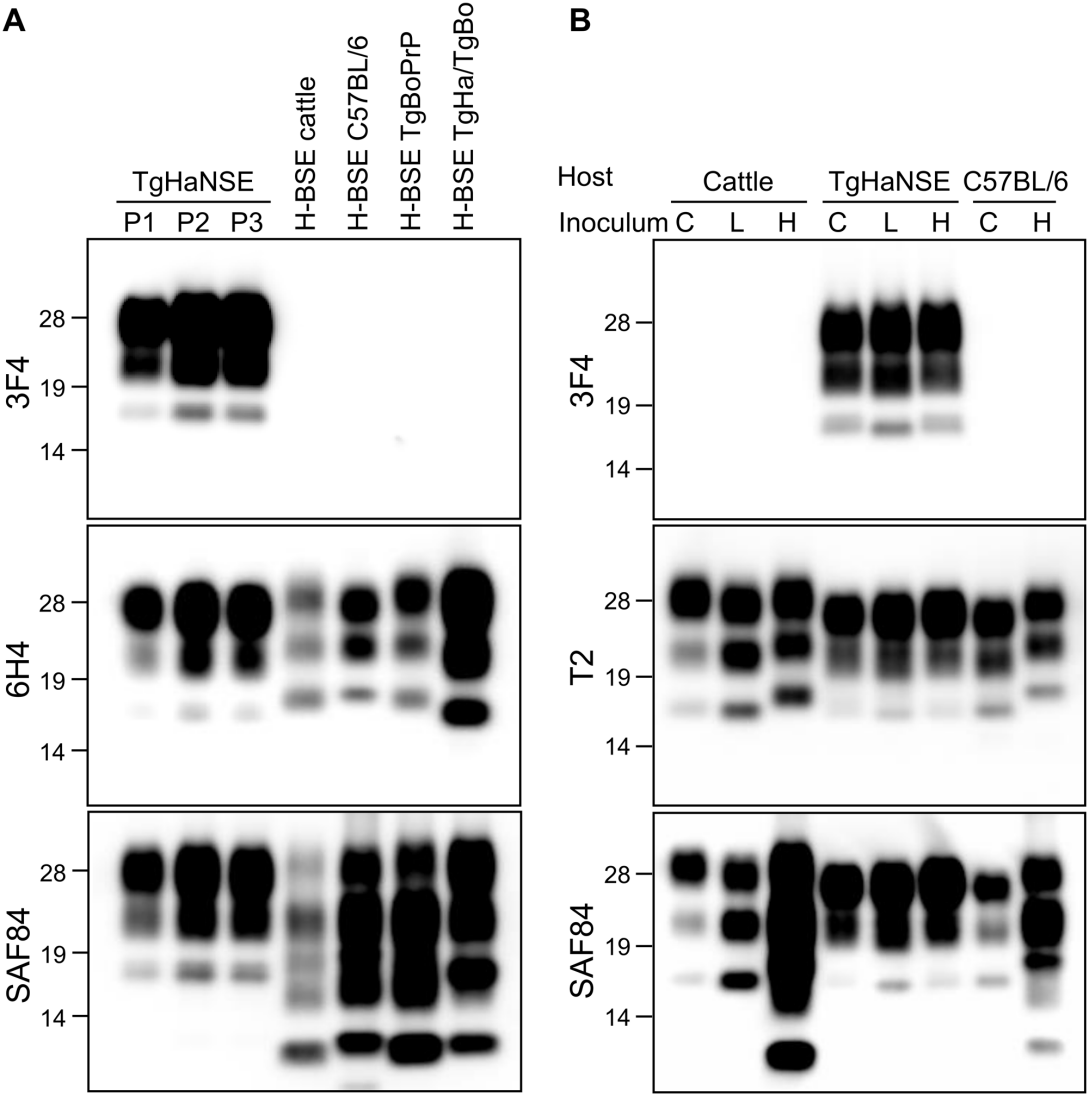
PrP^res^ profiles of H-BSE prions analyzed with mAbs 3F4, 6H4, T2, and SAF84. (A) Triple bands observed for TgHaNSE mice inoculated with H-BSE were smaller than those of cattle, C57BL/6 mice, and TgBoPrP mice analyzed with mAbs 3F4 and 6H4. TgHaNSE mice infected with H-BSE did not exhibit the 10–12-kDa band detected in cattle, C57BL/6 mice, and TgBoPrP mice infected with H-BSE, which were analyzed with the mAb SAF84. P1, P2, and P3, first, second, and third passage, respectively. TgHaNSE mouse-passaged H-BSE in TgBoPrP mice (H-BSE/TgHa/TgBoPrP) showed the molecular signatures of the H-BSE prion. (B) H-BSE (H), C-BSE (C), and L-BSE (L) strains showed similar molecular features in TgHaNSE mice. Molecular markers are shown to the left (kDa).

**Fig 5:**
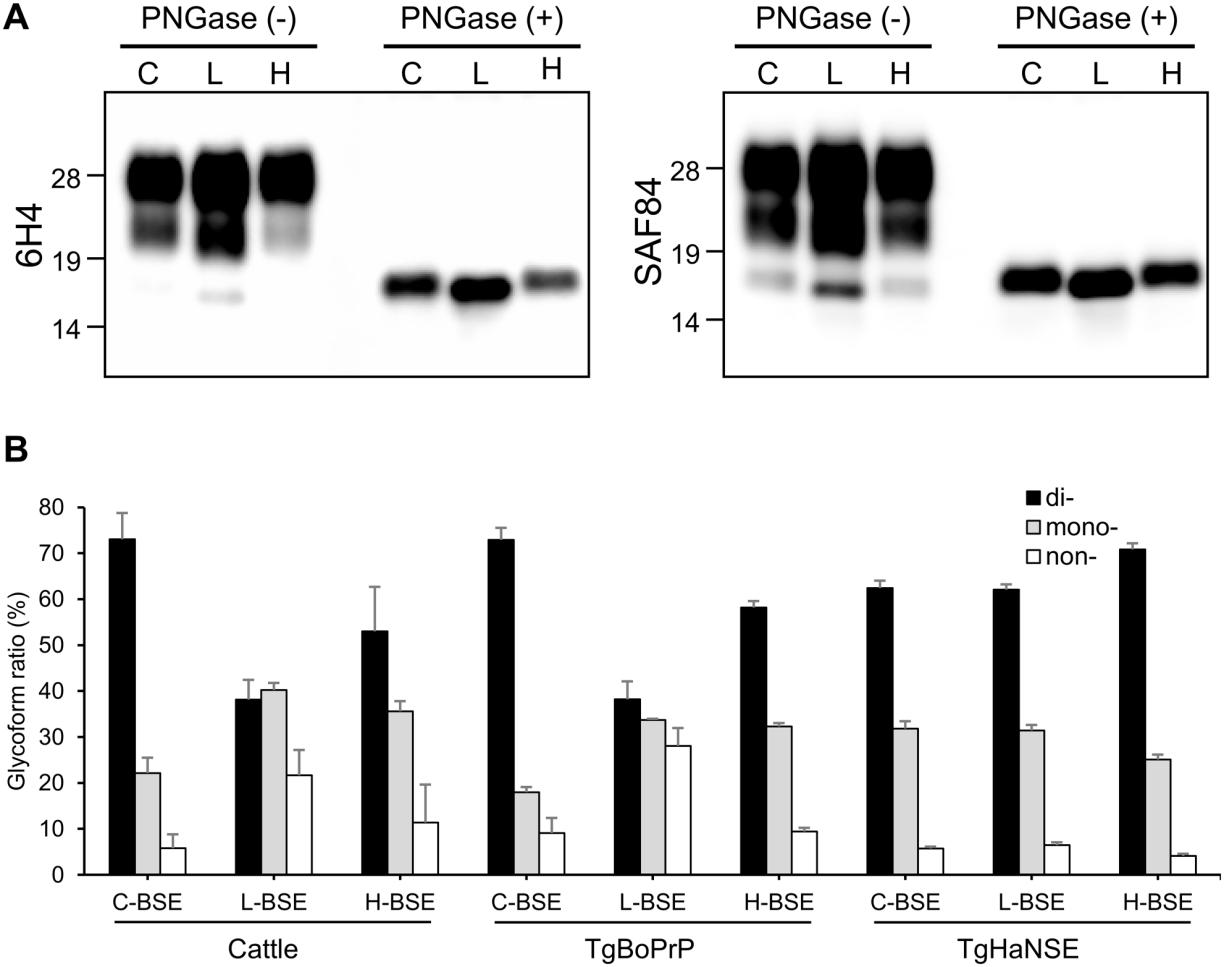
Molecular typing of PrP^res^ in brains of third-passage, H-BSE-infected TgHaNSE mice. (A) PrP^res^ expression was analyzed with the mAbs 6H4 and SAF84 before or after PNGase F deglycosylation. The unglycosylated fragment from TgHaNSE mice inoculated with H-BSE was similar in size to that of C-BSE and higher than that of L-BSE. Molecular markers are shown to the left (kDa). (B) PrP^res^ glycoform percentages in C-BSE-, L-BSE-, and H-BSE-infected mice were analyzed with the mAb T2. Glycoform ratios were similar between TgHaNSE mice inoculated with H-BSE and those inoculated with C- or L-BSE. Results are shown as mean ± standard deviation of triplicate experiments. Bar graph shows diglycosylated (white columns), monoglycosylated (gray columns), and unglycosylated (black columns) forms of the protein.

PrP^res^ signals in the brains of first- and subsequent-passage TgHaNSE mice infected with H-BSE showed similar molecular mass and glycoform patterns with mAbs 3F4, T2, 6H4, and SAF84 (Figs 4 and 5). There were no signals detected using the mAb 4E10 in TgHaNSE mice infected with three different BSE agents. In addition, TgHaNSE mice infected with H-BSE did not show the multiple banding patterns of PrP^res^ #1 and PrP^res^ #2 and lacked the 10–12 kDa band, which are generally observed for cattle and wild-type mice infected with H-BSE (Fig 4) [35]. The molecular mass of the unglycosylated form of PrP^res^ in the brains of H-BSE-infected TgHaNSE mice was similar to that of C-BSE and higher than that of L-BSE before and after deglycosylation with PNGase F (Fig 5A). Interestingly, the three fragments from TgHaNSE mice had lower molecular weights and reduced amounts of unglycosylated and monoglycosylated PrP^res^, whereas the amount of diglycosylated PrP^res^ was increased in TgHaNSE mice as compared to the original isolates from cattle and wild-type mice infected with H-BSE (Figs 4 and 5). The relative amounts of di-, mono-, and unglycosylated PrP^res^ in TgHaNSE mice infected with H-BSE were similar to those in mice infected with C- and L-BSE, which predominantly contained the diglycosylated form (Fig 5B). The molecular features of PrP^res^ were conserved in TgHaNSE mice between passages.

### Reverse transmission of H-BSE from TgHaNSE to TgBoPrP mice

**Fig 6:**
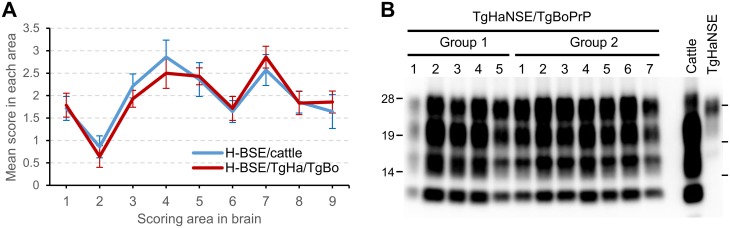
Vacuolar lesion scores and molecular features of TgBoPrP mice inoculated with H-BSE prions passaged in TgHaNSE mice. (A) Lesion profiles of first-passage TgBoPrP mice inoculated with H-BSE from cattle (H-BSE/cattle; blue), and H-BSE passaged once in TgHaNSE mice (H-BSE/TgHa/TgBo; red). Vacuolation was scored on a scale of 0–5 in the following brain areas: 1, dorsal medulla; 2, cerebellum; 3, midbrain; 4, hypothalamus; 5, thalamus; 6, hippocampus; 7, septal nuclei of the paraterminal body; 8 caudal cerebral cortex; and 9, rostral cerebral cortex. Data represent mean ± standard deviation (*n* = 6). (B) TgBoPrP mice inoculated with H-BSE passaged in TgHaNSE mice (TgHaNSE/TgBoPrP) yielded an additional fragment representing the traceback when probed with the SAF84 mAb. TgBoPrP mice were subdivided into two groups (*n* = 5 and 7) and inoculated on different days with the same brain homogenate prepared from first-passage TgHaNSE mice infected with H-BSE. Cattle, cattle infected with H-BSE; TgHaNSE, third-passage TgHaNSE mice infected with H-BSE. Molecular markers are shown to each side (kDa).

Within 30 days of transmission and directly before disease-related death, all TgBoPrP mice inoculated with H-BSE from TgHaNSE mice developed the typical clinical signs of the disease, including constant chewing of bedding, and were indistinguishable from TgBoPrP mice inoculated with H-BSE from cattle [13]. The mean survival period of 333.3 ± 19.3 days at primary passage (*n* = 12) was similar to that of H-BSE from cattle (i.e., the original isolate) (315.8 ± 11.6 days, *n* = 10) [13]. Pathological changes including lesion scores, PrP^Sc^ distribution patterns, and molecular features of PrP^res^ in the brains of TgBoPrP mice inoculated with H-BSE from TgHaNSE mice were identical to those in TgBoPrP mice inoculated with H-BSE from cattle (Fig 6A). The most conspicuous microscopic change in H-BSE-infected TgBoPrP mice was the presence of amyloid plaques along the periventricular and subcallosal regions [13]. The molecular properties of PrP^res^ in the brains of infected TgBoPrP mice were similar to those of cattle by WB analysis, which revealed the presence of an additional 10–12 kDa fragment using the mAb SAF84 (Fig 6B).

## Discussion

PrP^Sc^ of H-BSE from cattle was propagated with hamster PrP^C^ substrate in TgHaNSE mouse brains, as determined by WB and IHC analyses. These results demonstrate the transmissibility of H-BSE across a species barrier. Adaptation of prions to a new host requires several sequential passages. Third-passage mice showed the same shortened survival periods, characteristics of prion disease, and pathological and molecular features of PrP^Sc^ in the brain as second-passage mice. These data indicate that the infectivity and virulence of H-BSE increased in the new species during adaptation [36]. This was evidenced by the stability of H-BSE in the brains of TgHaNSE mice, which suggested that it had already adapted to the new host at the second passage [37]. After adaptation, the survival period of TgHaNSE mice infected with H-BSE was similar to that of TgBoPrP mice [13].

Differences in amino acid sequence between host PrP^C^ and PrP^Sc^ may create a species barrier that influences interspecies transmission [22]. Only eight amino acids differ between mouse and hamster PrP. Residue 155 in the hamster PrP sequence may affect the efficiency with which PrP^Sc^ is converted by hamster PrP^C^ and may explain the species barrier with respect to C-BSE prion transmission [38]. Plausible explanations for the efficient transmission of H-BSE to TgHaNSE mice is the high PrP^C^ expression level, host genetic factors, or the host microenvironment, which may contribute to the adaptation of heterologous prions and their propagation [36, 39, 40]. Therefore, under appropriate conditions such as overexpression of heterologous hamster PrP, the barrier might be overcome, enabling interspecies transmission and inducing PrP^Sc^ aggregation before primary passage TgHaNSE mice reach the end of their lifespan [41].

Although PrP^res^ from different species cannot be directly compared for strain characterization, the WB analysis revealed different molecular features for PrP^res^ in TgHaNSE mice as compared to the original H-BSE isolate from cattle, including differences in PK cleavage sites, glycoform patterns, and molecular weight. Interestingly, H-BSE-infected TgHaNSE mice did not show an additional 10–12-kDa band when analyzed using C-terminal-specific antibodies such as SAF84, which is typical in cattle and bovine transgenic or wild-type mice infected with H-BSE [6, 11–14, 19, 20, 35]. Furthermore, the relative amounts of PrP^res^ glycoform in TgHaNSE mice were altered relative to those in the original cattle inoculum. Although the glycoprofiles of TgHaNSE mice infected with H-BSE as compared to C- or L-BSE were similar, the unglycosylated molecular mass of PrP^res^ from L-BSE was less than that of PrP^res^ from H- and C-BSE. In addition, immunolabeled PrP^Sc^ distribution patterns in the brain differed for the three BSE agents in TgHaNSE mice. However, alterations in neuropathological phenotype and biochemical characteristics may be a general phenomenon in interspecies transmission [42, 43]. These results suggest that the glycoform of PrP^res^ in TgHaNSE mice may be influenced by characteristics of the host species rather than those of the prion strain [25, 44, 45]. The shift in the size of PrP^res^ has been reported in the transmission from sporadic Creutzfeldt-Jakob disease (CJD) to humanized transgenic mice [46], variant CJD to wild-type mice [47], and hamster Sc237 to wild-type mice [48]. These conformational transitions may be explained by the adaptation to the new host PrP^C^ and/or selection of a PrP^Sc^ subpopulation from heterogeneous PrP^Sc^, resulting in the emergence of a new prion strain [46].

The origin of prions that emerged in TgHaNSE mice inoculated with H-BSE was investigated in reverse transmission studies of H-BSE inoculum after passage from TgHaNSE to TgBoPrP mice, which revealed H-BSE-like phenotypes, a phenomenon known as traceback [49]. This indicated that the complex molecular features of H-BSE had reverted in H-BSE-infected TgBoPrP mice during back transmission. Several wild-type [19, 20] and bovine PrP transgenic mice [50] exhibited indistinguishable phenotypic features when inoculated with H-BSE as compared to C-BSE. However, transmission studies have revealed that H-BSE features are maintained in most wild-type [19, 20] and bovine transgenic mice [13, 16, 50]. Therefore, further back transmission studies will be useful for characterizing the changes from H- to C-BSE-like features during cross-species transmission.

In this study, we investigated PrP^Sc^ under artificial conditions, that is, by overexpressing transgenes in a heterogeneous environment. Nonetheless, our results provide a better understanding of how interspecies transmission of prion agents contributes to PrP^Sc^ diversity in prion pathogenesis. Additional studies are needed to clarify the transmissibility of H-BSE from TgHaNSE mice to hamsters and to examine PrP^Sc^ of H-BSE in TgHaNSE mice, as well as the influence of environment on its propagation in hamster brains.

In conclusion, H-BSE from cattle was efficiently transmitted to TgHaNSE mice, as determined by the accumulation of PrP^Sc^ in the brain as well as several peripheral tissues. Furthermore, H-BSE adapted and was stabilized after the third passage. The pathogenic features of H-BSE in TgHaNSE mice differed from those of C- and L-BSE. This mouse model is a useful tool for further investigations of the mechanisms underlying the species barrier of BSE prions.

## Supporting Information

S1 Fig Extracerebral PrP^Sc^ accumulation in third-passage TgHaNSE mice detected with mAb SAF84. (A) Coarse particulate PrP^Sc^ mainly accumulated in the gray matter of the spinal cord. (B) Fine punctate to coarse particulate PrP^Sc^ deposits were present in the ganglion cell layer, inner nucleus layer, and inner and outer plexiform layers of the retina. NFL, nerve fiber layer; GCL, ganglion cell layer; IPL, inner plexiform layer; INL, inner nucleus layer; OPL, outer plexiform layer; ONL, outer nucleus layer; PL, photoreceptor layer; RPE, retinal pigment epithelium. (C, D) Granular PrP^Sc^ immunoreactivity was observed in ganglionic cells (arrows) of the trigeminal ganglion (C) and dorsal root ganglion (D). (E) Granular PrP^Sc^ immunoreactivity was detected in the intrafusal myofibers of muscle spindles of skeletal muscle. (F) Serial section of (E) with HE staining.
(PDF)
