# Supplementary figures and images for: Transmissibility of H-Type Bovine Spongiform Encephalopathy to Hamster PrP Transgenic Mice
Source: PLoS One. 2015 Oct 14;10(10):e0138977. doi: 10.1371/journal.pone.0138977 (PMC4605493; doi:10.1371/journal.pone.0138977)

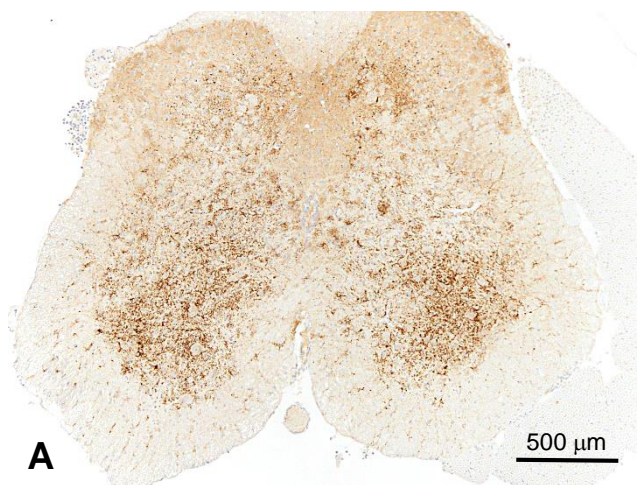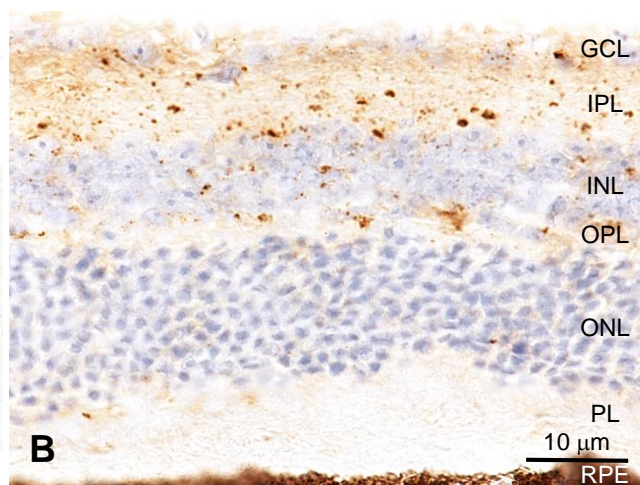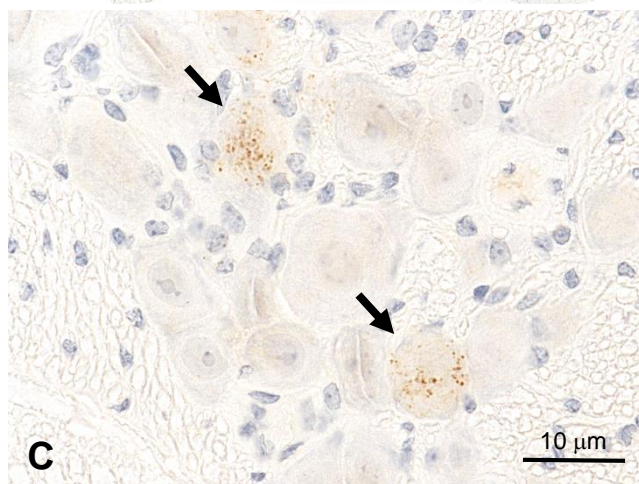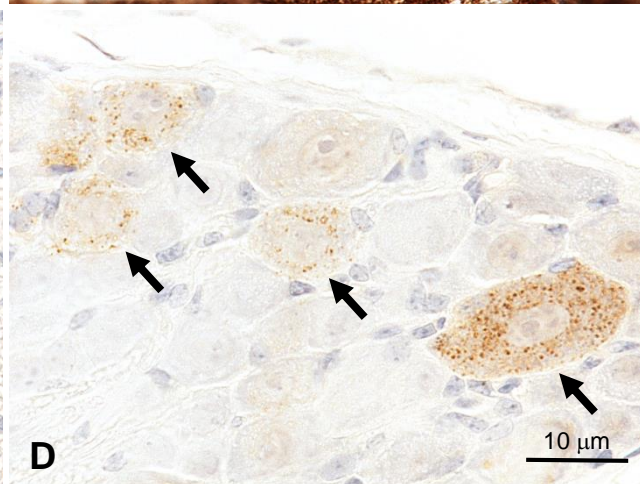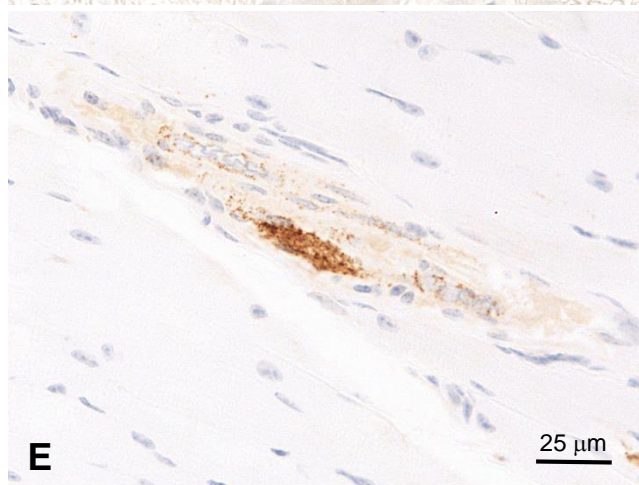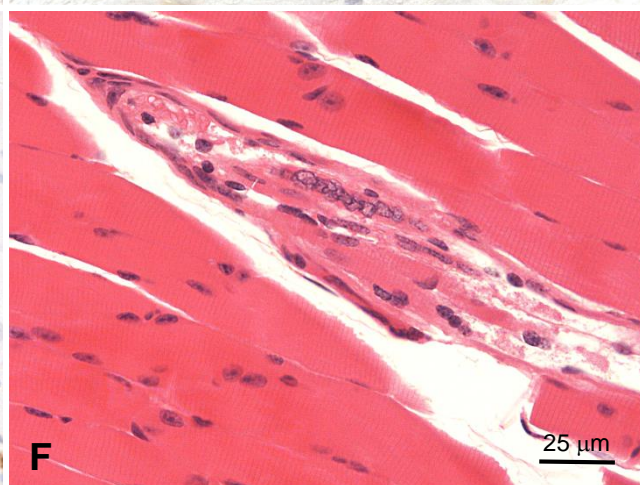

Supplement: S1 Fig — (A) Coarse particulate PrPSc mainly accumulated in the gray matter of the spinal cord. (B) Fine punctate to coarse particulate PrPSc deposits were present in the ganglion cell layer, inner nucleus layer, and inner and outer plexiform layers of the retina. NFL, nerve fiber layer; GCL, ganglion cell layer; IPL, inner plexiform layer; INL, inner nucleus layer; OPL, outer plexiform layer; ONL, outer nucleus layer; PL, photoreceptor layer; RPE, retinal pigment epithelium. (C, D) Granular PrPSc immunoreactivity was observed in ganglionic cells (arrows) of the trigeminal ganglion (C) and dorsal root ganglion (D). (E) Granular PrPSc immunoreactivity was detected in the intrafusal myofibers of muscle spindles of skeletal muscle. (F) Serial section of (E) with HE staining. (PDF) [file pone.0138977.s001.pdf]
